# Improving Agricultural Product Traceability Using Blockchain

**DOI:** 10.3390/s22093388

**Published:** 2022-04-28

**Authors:** Qi Yao, Huajun Zhang

**Affiliations:** School of Computer Science and Artificial Intelligence, Changzhou University, Changzhou 213164, China; 17315383917@163.com

**Keywords:** blockchain, traceability, agricultural products, Ethereum, IPFS, smart contract

## Abstract

Most traditional agricultural traceability systems are centralized, which could result in the low reliability of traceability results, enterprise privacy data leakage vulnerabilities, and the generation of information islands. To solve the above problems, we propose a trusted agricultural product traceability system based on the Ethereum blockchain in this paper. We designed a dual storage model of “Blockchain+IPFS (InterPlanetary File System)” to reduce the storage pressure of the blockchain and realize efficient information queries. Additionally, we propose a data privacy protection solution based on some cryptographic primitives and the Merkle Tree that can avoid enterprise privacy and sensitive data leakage. Furthermore, we implemented the proposed system using the Ethereum blockchain platform and provided the cost, performance, and security analysis, as well as compared it with the existing solutions. The results showed that the proposed system is both efficient and feasible and can meet the practical application requirements.

## 1. Introduction

Agricultural products are highly prized for their freshness, health, and nutritional value. Simultaneously, chlorophyll is abundant in agricultural products, which promotes metabolism and alleviates fatigue. However, because agricultural products’ preservation and storage requirements, as well as their transportation requirements, are more stringent, agricultural safety accidents frequently occur [1].

Agricultural product safety incidents put people’s dietary safety and health at risk, which causes a major crisis in consumer trust in the agricultural industry. As a result, countries around the world have started to value agricultural product supply chain traceability and have passed laws and regulations to improve agricultural product traceability management. China’s 2009 Food Safety Law requires that food producers use information technology to keep track of production and operation data and set up a food safety traceability system [2]. The European Union’s General Food Law, enacted in 2002, requires the food industry to establish a comprehensive traceability system that enables timely and accurate recall targets and information delivery to consumers [3].

Traceability has developed into a critical component of the agricultural supply chain. The traceability of agricultural products refers to the process of tracing all links in the agricultural product’s supply chain [4]. The agricultural product traceability system records the key details of the entire process of agricultural products from production to the table. Through the agricultural product traceability system, consumers can obtain information about the source of agricultural products they consume, and regulators can quickly identify problematic agricultural product links, identify responsible parties, recall problematic products in a timely manner, and minimize losses to the greatest extent possible [5]. However, the supply chain for agricultural products is complex, making agricultural safety supervision and traceability particularly challenging in practice. The majority of existing traceability systems use a centralized architecture that is managed and maintained by a third party, such as a business or government agency [6,7]. This results in issues such as insecure data storage, low traceability reliability, single-point attack vulnerability, and data privacy [8].

Trusted traceability means the security, integrity, availability, accountability, and nonrepudiation of traceability information can be ensured, and the serious trust problem caused by centralized, monopolistic, asymmetric, and opaque agricultural product supply chains can be solved. How to achieve trusted traceability for agricultural products has attracted increasing attention from academics and practitioners [9]. A potential solution to achieve the above goal is blockchain technology, which can ensure data integrity and prevent tampering and any single-point failure [10]. Blockchain technology has gained widespread attention as a result of the emergence and popularity of cryptocurrencies such as Bitcoin [11]. Blockchain is a decentralized, tamper-resistant, and traceable distributed database that utilizes a cryptographic algorithm to create a chain structure composed of chronologically ordered blocks of data [12,13,14]. Through distributed data storage, encryption algorithms, peer-to-peer transmission, and other technical support, blockchain technology can ensure the secure storage of traceability data and the nonrepudiation of information sources, enabling agricultural products to have trusted traceability [15,16].

Blockchain can provide a secure access environment for the large amount of data generated by sensors used within the agricultural supply chain [17,18]. However, blockchain technology faces new technical challenges, including transaction processing capacity and data explosion [19]. As a result, processing and storing agricultural product traceability information directly on the blockchain is challenging. In addition, because all data on the blockchain are public and transparent, this could lead to private and sensitive enterprise information leaking.

Therefore, this paper proposes an agricultural product traceability system based on the Ethereum blockchain to ensure the security, traceability, immutability, and accessibility of data provenance for agricultural products. In this paper, we focused on the storage capacity and scalability issues and privacy leakage, which are the primary issues confronting the agricultural product traceability system. First, we designed a dual storage model based on blockchain and the InterPlanetary File System (IPFS), which alleviates blockchain’s storage pressures, increases query speed, and increases system flexibility. Second, by leveraging some cryptographic primitives and the Merkle Tree, we proposed a data privacy protection solution for traceability information to avoid the disclosure of enterprises’ sensitive data (e.g., specific transaction information). Third, we implemented and tested the proposed traceability system and conducted detailed cost, performance, and security analysis. Finally, we compared the proposed system with the existing work and discussed the limitations of the proposed system and future research directions.

## 2. Background and Related Work

### 2.1. Blockchain

Blockchain is a decentralized ledger that stores transaction information in blocks and connects all blocks via a chain [20]. Blockchain technology originated with a 2008 paper titled “Bitcoin: a peer-to-peer electronic cash system” published by an academic named Nakamoto [21]. A blockchain network does not require a trusted central server and can be run decentralized [22]. It is available to anybody, and all nodes in the distributed peer-to-peer network share the same data, verifying transactions according to a consensus mechanism [23,24]. Therefore, blockchain has the characteristics of immutability, transparency, and trustworthiness [25,26]. Additionally, blockchains can be classified as public blockchains, private blockchains, or consortium blockchains depending on some principles, such as the authentication and access control techniques [27].

### 2.2. Smart Contract

The concept of smart contracts, introduced by Nick Szabo in 1997 [28], has gained popularity with the rapid development of blockchain technology [29,30]. A smart contract is a self-executable, self-enforceable, self-verifiable, and self-constraining piece of source code stored on the blockchain [31,32,33]. Ethereum was the first blockchain platform that introduced smart contracts [34]. Ethereum supports the Turing-complete scripting language, which allows writing smart contracts with complex logic. Ethereum’s core is the Ethereum virtual machine (EVM), which can execute complex code on the blockchain.

### 2.3. IPFS

The Interplanetary File System (IPFS) is a peer-to-peer distributed file system where data are stored in the form of chunks [35]. Any node in an IPFS network is independent and does not need to trust the others, so there is no single point of failure as in traditional HTTP (Hyper Text Transfer Protocol) transmission [36]. When a user stores a file in IPFS, IPFS generates a 32-bit hash as a result of data storage. The peer-to-peer transmission of IPFS can significantly reduce network bandwidth consumption, and distributed files can significantly reduce the risk of DDoS (Distributed denial of service) attacks [22].

### 2.4. Related Work

Traceability refers to the ability to obtain any or all information throughout its entire life cycle using recorded identifications [37]. Traceability enables users to track agricultural products throughout their lifecycle, from raw ingredients to manufacturing, processing, shipping, and consumption [38]. For agricultural products, if a safety or quality incident occurs, the supply chain link causing the problem can be swiftly identified, blame can be assigned, and targeted punitive measures can be executed. As a result, building a traceability system for agricultural products is an effective way to make sure that agricultural products are safe and to build trust between agricultural producers and their customers.

Blockchain technology has the characteristics of being data tamper-proof, distributed, decentralized, and traceable, which provides a possible solution to the traditional agricultural product traceability system [39]. In recent years, more and more scholars have conducted exploratory research on the application of blockchain technology in traceability scenarios. Table 1 provides a critical analysis of the existing works in the literature.

Zhao et al. reviewed existing work on blockchain technology in agri-food value chain management and summarized the key challenges, mainly including storage capacity and scalability issue, privacy leakage, regulation problems, high cost problem, throughput and latency issue, and lack of skills [40]. Feng Tian proposed an agri-food supply chain traceability system based on RFID and Blockchain technology, which enhanced the reliability of agri-food traceability information [41]. However, the system has the issues of high cost, data privacy leakage, and storage capacity issue. Liu et al. proposed an RFID-based blockchain big data traceability security model to achieve secure RIFD big data traceability management [42]. This scheme is at the stage of theoretical analysis and has not been implemented and tested on a specific blockchain platform.

**Table 1:** Critical analysis of the existing works in the literature.

Author	Year	Objective	Technologies	Merits	Demerits
Tian [41]	2016	To use blockchain combined with RFID for building the agri-food supply chain traceability system.	RFID, Blockchain	Enhance the reliability of the agri-food traceability information.	High cost for using RFID; data privacy leakage; Poor system storage capacity issue
Liu et al. [42]	2018	Proposed a Security Provenance Model for RFID Big Data Based on Blockchain	Blockchain, RFID big data	Applying blockchain technology in the process of tracking and tracing IoT big data.	No detailed experimental process and analysis process.
Lin et al. [43]	2019	Proposed a food safety traceability system based on blockchain and EPCIS	Ethereum, EPCIS and Smart Contract	Collaborative management model of on-chain and off-chain data	System performance is limited by the amount of data.
Baralla et al. [44]	2019	Proposed a generic agri-food supply chain traceability system based on blockchain technology.	Hyperledger Sawtooth, Smart contract	Eliminate the centralization of information in the supply chain.	The issue of enterprise data privacy leakage; Low maturity of the Sawtooth platform.
Chen et al. [45]	2021	Designed a food traceability system based on blockchain	Ethereum, Smart Contract	Detailed system design and traceability process	No traceability information privacy protection
Dey et al. [46]	2021	Proposed a Blockchain and QR-code-based framework to digitize food production information and retrieval.	Blockchain, QR Code, and Cloud computing	Offer flexible scalability and improve the storage capacity.	The framework need more computationally powerful cloud server as the number of products grows.
Dey et al. [47]	2022	Proposed a blockchain-based framework to reduce food waste in a Web 3.0-enabled smart city.	Machine Learning, Blockchain, Cloud Computing, and QR Code	Use several cut-ting-edge technologies in conjunction to reduce food waste efficiently.	Lacks in showing the specific implementation details

Lin et al. proposed and implemented a food safety traceability system based on blockchain and EPCIS and adopted the dynamic management of on-chain and off-chain data to solve data explosion problems on the blockchain [43].

Baralla et al. proposed a generic agri-food supply chain traceability system based on Hyperledger Sawtooth [44]. Consumers can easily access traceable and verifiable product information by using QR codes. However, privacy data protection was not discussed in the system, and the maturity of the Sawtooth platform is relatively low.

Chen et al. designs a food traceability system based on the Ethereum platform and devises a dual storage model to store the complete data in the local database and the hash value of traceability information in the blockchain, thus improving the operational efficiency of the blockchain and solving the scalability problem of the blockchain [45].

Dey S et al. proposed a blockchain and QR (Quick Response)-code-based framework for digitizing food production information and retrieval, thereby making it easily accessible, traceable, and verifiable by consumers and producers [46]. The proposed framework was implemented at a large scale in the cloud, which can improve the storage capacity of blockchain and offer flexible scalability as per the consumer’s demand. However, if the farm produces more than 10,000 items per day, the framework will require a more powerful cloud server, which may result in increased costs. Dey et al. used several cutting-edge technologies, including blockchain technology, cloud computing, QR codes, and reinforcement learning in conjunction to develop a framework that could reduce food waste efficiently [47].

However, the previously mentioned studies are still not perfect in terms of storage capacity and scalability issues, and the risk of enterprise sensitive data leakage is still present. Our study designs a dual storage model to alleviate blockchain’s storage pressures, increase query speed, and improve system flexibility. Additionally, we propose a data privacy protection solution to prevent enterprise sensitive information disclosure during information interaction among stakeholders. Finally, we implement and test the proposed traceability system and conduct a detailed analysis.

## 3. Research Design

**Figure 1:**
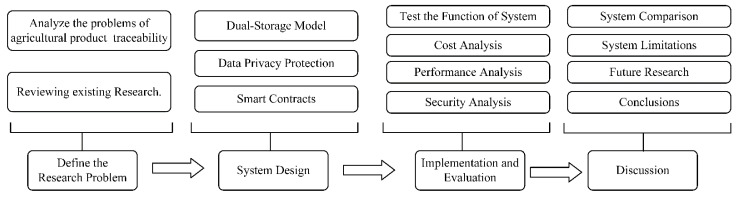
The flow of research stages.

The goal of our research is to improve agricultural product traceability using blockchain. Through research and comparison of related work, the agricultural traceability system still faces storage capacity issues and privacy leakage. This paper focuses on these key problems and conducts deeper research. As shown in Figure 1, our research is divided into four main stages, namely: Define the Research Problem, System Design, Implementation and Evaluation, and Discussion.

The first stage of our research is to define the research problem. First, we carefully analyzed the current agricultural product traceability system. Second, we discussed some of the related work in traceability using blockchain technology. Finally, we defined the research problem as how to build a reliable agricultural traceability system that solves storage capacity issues and privacy leakage.

The second stage of the research is system design. To solve the research problems, we proposed a dual storage model of “Blockhain+IPFS” and a data privacy protection solution based on some cryptographic primitives and the Merkle Tree. Additionally, the smart contracts of the proposed system are designed in detail.

The third stage is system implementation and evaluation, which aims to test and validate key functions of the system. At this stage, we performed a specific analysis of the system, focusing on cost, performance, and security. In the cost analysis, the gas costs of different function calls of smart contracts are analyzed. In the performance analysis, the proposed data privacy protection solution, as well as the query and upload times of different file sizes, are tested and analyzed. In the security analysis, we analyzed the security of the system from the following perspectives: data integrity, availability, accountability, and authorization.

The last stage is the discussion of the proposed system. At this stage, the proposed system is compared to the traditional centralized agricultural traceability system and related work. Additionally, the limitations of the system are discussed in detail, and future research directions are provided.

## 4. System Design

### 4.1. System Architecture

**Figure 2:**
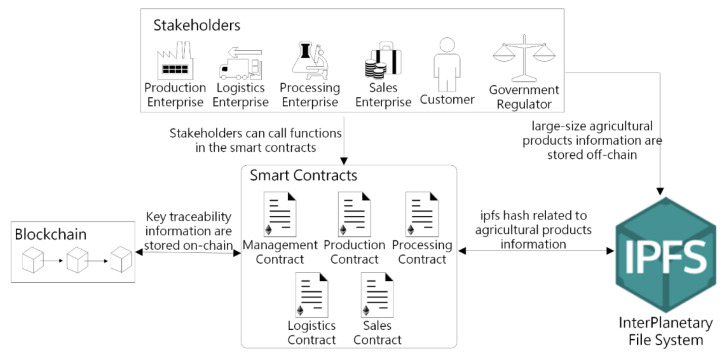
A high-level architecture for the proposed blockchain-based agricultural traceability system.

We propose an Ethereum blockchain-based system for agricultural product traceability to accurately record, share, and trace the specific data within the whole supply chain. Our system leverages blockchain technology to increase transparency, foster trust, and strengthen information security among agricultural product supply chain players. Additionally, our system develops smart contracts and uses IPFS decentralized storage technology to enhance the reliability of traceability results and system flexibility. A high-level architecture for the proposed agricultural traceability system together with the stakeholders and their interactions with the system was shown in Figure 2.

**Figure 3:**
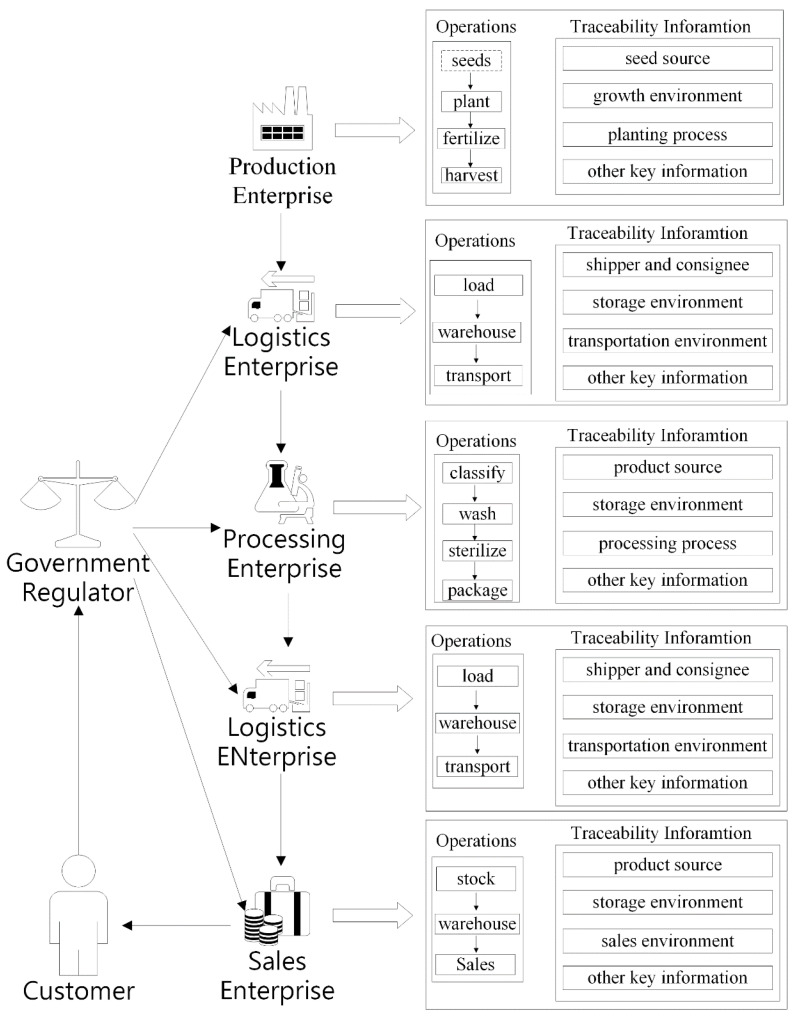
The agricultural supply chain process.

The stakeholders of the agricultural product traceability system can be divided into three types: enterprises, consumers, and government regulators. Enterprises primarily include production enterprises, processing enterprises, logistics enterprises, and sales enterprises, which correspond to the production, logistics, processing, and sales links of the agricultural supply chain. The agricultural supply chain process is shown in Figure 3, and the roles and responsibilities of each enterprise are described below. As the origin of the agricultural supply chain, production enterprises are primarily responsible for producing raw materials for agricultural products, which includes planting, watering, fertilizing, monitoring for pests and diseases, picking and bagging, and so on. It is important for production enterprises to keep track of things such as temperature, humidity, and light at each link of agricultural products’ growth and maturity, as well as the use of chemical fertilizers and pesticides. Processing enterprises buy raw agricultural products from production enterprises and process them, which may include sorting, washing, cutting, peeling, sterilizing, fermentation, distillation, decolorization, and packaging. Finally, processing enterprises convert agricultural raw materials into marketable agricultural products. 

Since they are responsible for agricultural product transportation, logistics enterprises track agricultural product logistics information in real time via positioning systems and video monitoring systems to avoid commodities being switched and substandard goods being used as substandard goods during the logistics process. Due to the nature of agricultural products, such as their inability to be stored and their susceptibility to corrosion, agricultural products frequently require cold chain transportation during the shipping process. As a result, logistics firms must track the shipping environment, including temperature and humidity, in order to assure the freshness and safety of agricultural products. Since sales enterprises are the final links in the agricultural supply chain, they sell agricultural products to consumers. Sales enterprises must maintain records of the agricultural products’ origin, the storage environment in which the products are stored, the sales staff, and transaction information.

The stakeholders can interact with the smart contracts to perform the corresponding functions through an Application Program Interface (API) such as Infura, Web3, and JSON RPC (JavaScript Object Notation Remote Procedure Call). Our proposed system consists of five smart contracts, and each smart contract is focused on a specific task. The management contract focuses on enterprise registration and management. The remaining four smart contracts focus on recording and querying traceability information during the agricultural product supply chain and updating the status of agricultural products to realize the whole process of agricultural product traceability. The database of this system includes IPFS and blockchain, where IPFS is responsible for the off-chain storage of detailed traceability information of agricultural products in each link, and blockchain stores small-size key information on-chain, which is used to check whether the traceability information has been tampered with.

### 4.2. Dual Storage Model and Privacy Protection Solution

Since the agricultural product supply chain involves many links, the IoT (Internet of Things) terminal devices at each link and the participating subjects will generate thousands or even terabytes of data in real time. Additionally, to improve the reliability and accuracy of agricultural product traceability, the data that need to be saved for traceability in each link need to be as detailed as possible. If all the data were stored in the blockchain, it would lead to a data explosion and poor system flexibility. Additionally, the data involved in the agricultural product supply chain contain some sensitive and private information that is viewable only by relevant enterprises, such as transaction information. The visibility of all members is a critical characteristic of the blockchain, which may lead to enterprise privacy data leakage.

**Figure 4:**
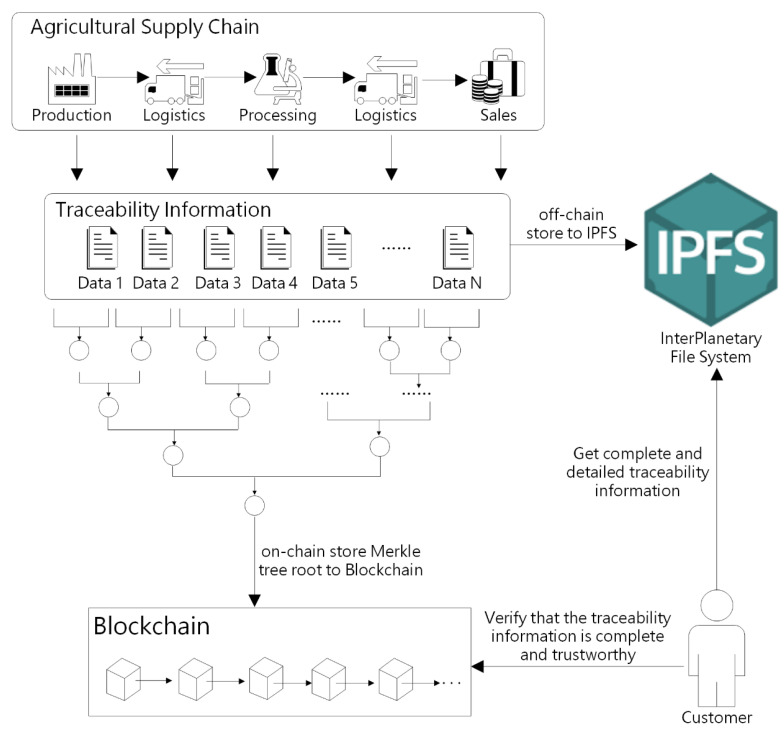
Dual storage model for traceability information.

Therefore, in order to solve the above-mentioned problems in the traceability of agricultural products, we use a novel dual storage model with data privacy protection. As shown in Figure 4, the main feature of this model is as follows: First, traceability information of agricultural product is split according to different links and different attributes. Then, a Merkel Tree is built with all the data contained in each link of traceability information, using cryptography to ensure the privacy and security of the data. Second, all attributes of traceability information of agricultural products at different links in the supply chain are stored off-chain into IPFS (InterPlanetary File System) separately. Third, the key traceability information is stored on-chain in order to reduce the amount of on-chain data.

With the model we designed, the system can ensure the integrity and authenticity of the traceability information and can also alleviate the risk of blockchain data explosion. Additionally, enterprises do not need to worry about data privacy issues, and they can achieve fine-grained traceability and information sharing. The detailed design of this model is described below. 

#### 4.2.1. Data Privacy Protection

According to Section 4.1, agricultural product traceability information can be divided into four categories according to different supply chain links. Suppose each link of agricultural product information contains N pieces of data, where N is a power of 2. We use $data_{i}$ to represent each datum. We then constructed these N data into a Merkle Tree, and the specific steps were as follows. 

First, each datum was calculated by SHA256 hash function to obtain N leaf nodes, and the value of $Node_{i}$ was $Hash_{i}=SHA256\left(data_{i}\right)$. Second, for neighboring nodes $Node_{1}$ and $Node_{2}$, their parent node $Node_{\left[1,2\right]}$ was generated upwards, and the value of $Node_{\left[1,2\right]}$ was $Hash_{\left[1,2\right]}=SHA256(Hash_{1}||Hash_{2})$. According to the above method, $Node_{\left[3,4\right]}$, $Node_{\left[5,6\right]}$, …, were continuously generated. Third, for neighboring nodes $Node_{\left[1,2\right]}$ and $Node_{\left[3,4\right]}$, their parent node $Node_{\left[1,4\right]}$ was generated upwards, and the value of $Node_{\left[1,4\right]}$ was $Hash_{\left[1,4\right]}=SHA256(Hash_{\left[1,2\right]}||Hash_{\left[3,4\right]})$. According to the above method, $Node_{\left[5,8\right]}$, $Node_{\left[9,12\right]}$, …, $Node_{\left[N-3,N\right]}$ were continuously generated. Finally, following the steps above, N leaf nodes were aggregated into one root node $Node_{\left[1,4\right]}$, and the value of $Node_{\left[1,4\right]}$ was $Hash_{\left[1,4\right]}=SHA256(Hash_{\left[1,N/2\right]}||Hash_{\left[N/2+1,N\right]}),N=4$.

**Figure 5:**
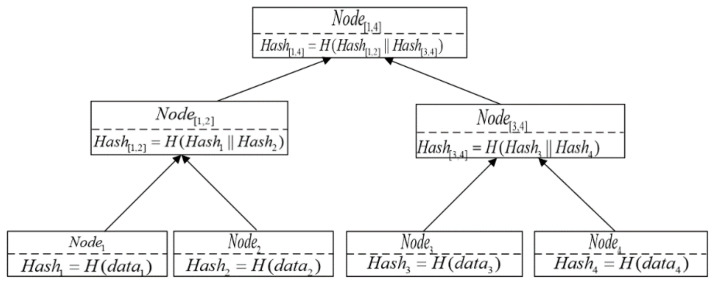
Merkle Tree for traceability information.

As shown in Figure 5, suppose there are four pieces of data in the traceability information of an agricultural product at a certain link. If $data_{2}$, $data_{3}$, and $data_{4}$ are sensitive and private data of the enterprise and the enterprise does not want it to be exposed to consumers or other enterprises, the enterprise only needs to present the following values: ($data_{1}$, $Hash_{2}$, $Hash_{\left[3,4\right]}$), and we call ($Hash_{2}$, $Hash_{\left[3,4\right]})$ the Merkle path of *data*_1_. The value of the Merkle root node can be obtained by continuously SHA256 (Secure Hash Algorithm 256) hashing the date and Merkle path.

Due to the hiding or one-way characteristic of the hash function, the computation process of the hash function is one-way irreversible, which ensures that the data content cannot be calculated by the hash value of the data. Furthermore, because of the collision resistance characteristic of the hash function, once the data provided by the enterprise to the consumer are tampered with or incomplete, the computed hash value must change, resulting in an inconsistent hash value for the constructed Merkle Tree root node.

#### 4.2.2. On-Chain Storage 

According to the solution in Section 4.2.1, the N data in each link of traceability information, after continuous SHA256 hash calculation, can finally construct a Merkle Tree. To ensure the integrity and reliability of agricultural product traceability information, the constructed Merkle Tree root must be safely stored on the blockchain so that consumers can verify the integrity and authenticity of the traceability information.

The key characteristic of blockchain technology is immutability, which refers to data that cannot be changed or altered. Therefore, we store the Merkle Tree root on the blockchain. Table 2 illustrates the on-chain storage format. The key information of each agricultural product includes 10 items, including the ID of the product, the state of the product, and the enterprise EA corresponding to the four main links of the supply chain, as well as the Merkle Tree root node constructed from the traceability information.

The Ethereum address is a hexadecimal number, an identifier derived from the last 20 bytes of the Keccak-256 hash of the public key. A Merkle Tree is constructed using SHA256 hash function, so the Merkle Tree root is 32 bytes. We use the UUID (Universally Unique Identifier) to generate a unique agricultural product ID, and each ID is 16 bytes. Therefore, the on-chain storage space required for each agricultural product in the blockchain is calculated to be 224 bytes, which is relatively small and acceptable.

#### 4.2.3. Off-Chain Storage 

IPFS is a distributed file system that uses a peer-to-peer network to store and share data. Every file stored on IPFS is hashed and associated with a unique resource address. Unlike a blockchain-based system, which can dump the chain to reveal all data, IPFS requires a unique resource address to locate and retrieve data via DHT (Distributed Hash Table) [22]. Therefore, we use the IPFS, a low-cost off-chain storage system, to store complete and detailed traceability information about agricultural products. 

According to the solution in Section 4.2.1, each link of agricultural product information is constructed into a Merkle Tree, and each piece of data corresponds to a Merkle path. We upload each datum and its Merkle path into IPFS and obtain the corresponding IPFS hash. Therefore, enterprises can share specified data in traceability information with users, which eliminates the issue of privacy leakage.

For example, the four data in Figure 4 are stored off-chain in IPFS, and the returned results are shown in Table 3. If only $data_{1}$ and $data_{2}$ can be shared with consumers, then the enterprise sends ipfs_hash_1 and ipfs_hash_2 to users in the form of QR codes, etc. The consumer has access to data_1 and data_2 contents via ipfs_hash_1 and ipfs_hash_2. In order to verify the integrity and authenticity of the data, the consumer will obtain the Merkle path and date in turn for SHA256 hash function calculation and finally calculate the value of the root node. In order to verify the integrity and authenticity of the data, the consumer first hashes the data with SHA256 and keeps hashing the calculated hash value with Merkle path to finally obtain the Merkle root. If the value is consistent with the Merkle Tree root stored on-chain in the blockchain, then it means that the data have not been tampered with.

### 4.3. Sequence of Operations

**Figure 6:**
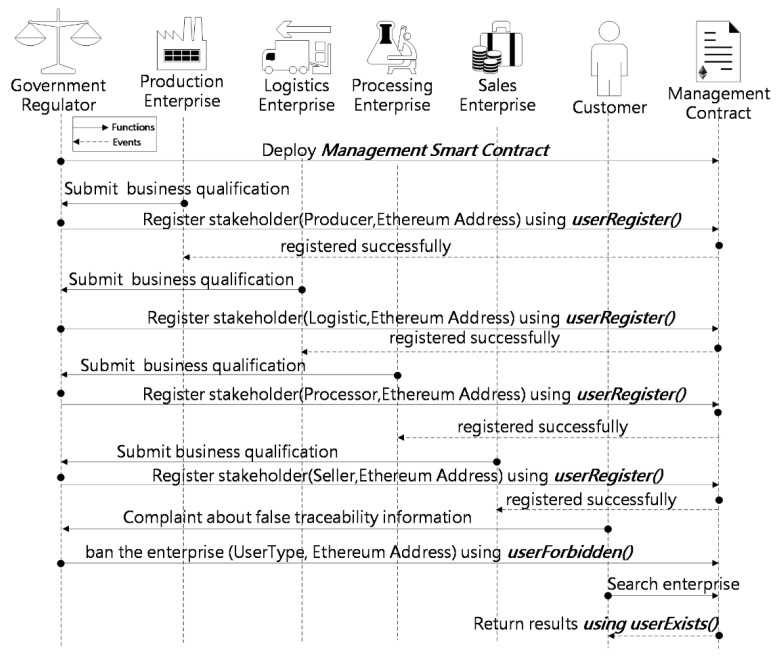
Sequence diagram showing interactions among the stakeholders with the Management smart contract.

The sequence diagrams of the traceability system’s main operations are presented in this subsection in the form of functions and events. Additionally, the sequence diagram illustrates the interaction of the various stakeholders with the smart contract. The sequence diagram in Figure 6 depicts the Management smart contract’s interaction with the government regulator, production enterprise, logistics enterprise, processing enterprise, sales enterprise, and customers. The government regulator initiates the system by deploying Management smart contract, which records all legitimate agricultural supply chain enterprises in the traceability system using the data type “mapping (address ≥ bool)”.

After the agricultural supply chain enterprise submits the necessary business licenses and qualifications to the government regulator and the audit is approved, the government regulator invokes the function *userRegister()* and enters the enterprise’s Ethereum Address (EA) and enterprise type to complete the registration. After registration is complete, the enterprise’s EA changes to “true” in the contract. Consumers can call function *userExists()* to check whether an enterprise is a legitimate enterprise of the traceability system. If the government regulator receives a complaint from consumers, after confirmation, it can call the function *userForbidden()* to block the corresponding enterprise’s account and restrict its access to the system, which means that the EA of the enterprise changes to “false” in the contract.

**Figure 7:**
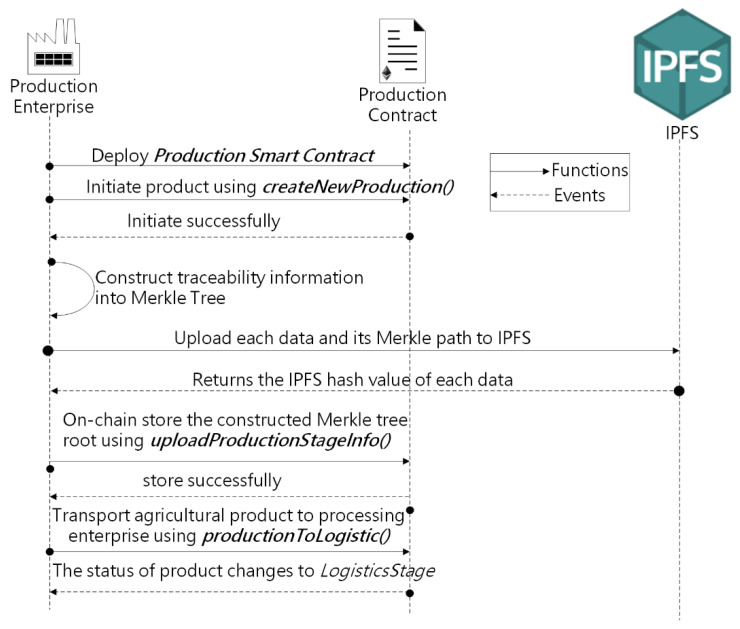
Sequence diagram showing interactions among the production enterprise with the Production smart contract.

The sequence diagram displayed in Figure 7 presents the interaction of the production enterprise with the Production smart contract. The production enterprise begins by deploying a Production smart contract, which is inherited from the government regulator’s Management contract. As the first link in the agricultural product supply chain, the production enterprise needs to initialize each product by calling the function *createNewProduction()*, which creates a structure for each agricultural product with 10 fields, as described in Table 1. Each agricultural product has five states in the traceability system, namely, *ProductionStage*, *LogisticsStage*, *ProcessStage*, *SaleStage*, and *Sold*. When the product has been initialized, the state of the agricultural product is *ProductionStage*. When the production enterprise completes the production of this agricultural product, it will store the traceability information of the agricultural product collected through the Internet of Things, etc., both on-chain and off-chain according to the method in Section 4.2.

Then, the production enterprise can call the function *uploadProductionStageInfo* to store the Merkle Tree root in the blockchain. When the production enterprise and the processing enterprise complete the transaction, the production enterprise can call the function *productionToLogistic* to ship the product to the processing enterprise, and the production enterprise needs to enter the EA of the logistics enterprise in this function. After the function is executed, the state of the produce changes to *LogisticsStage*. Like the production link of agricultural products, the corresponding enterprise is responsible for recording traceability information in the processing, logistics, and sales links of the agricultural supply chain. The status of the agricultural products will go through four states: *LogisticsStage*, *ProcessStage*, *SaleStage*, and *Sold*. When the state of the produce is *Sold*, it means that the agricultural product has been purchased by the consumer.

**Figure 8:**
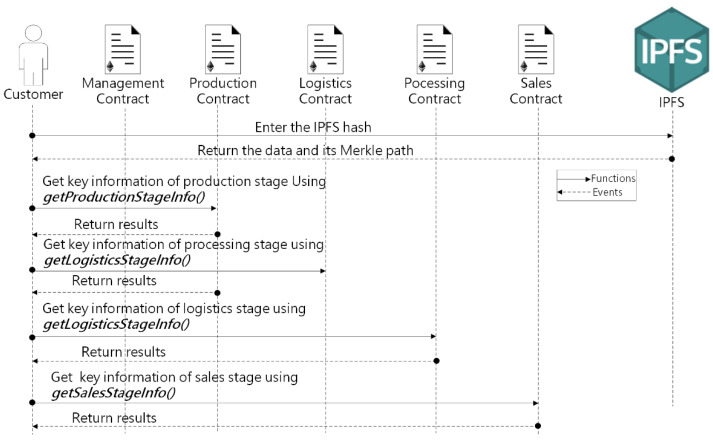
Sequence diagram showing interactions among the customers with the smart contracts and IPFS.

The sequence diagram displayed in Figure 8 presents the interaction of customers with the smart contracts and IPFS. Consumers can view and verify the traceability information of agricultural products by scanning QR codes and other means, and the specific steps are as follows: First, consumers enter the IPFS hash corresponding to the data they want to view in IPFS. Second, consumer download the data from IPFS and the corresponding Merkle path. Third, consumers construct a Merkle Tree root using the data and Merkle path. Fourth, consumers use the query function such as *GetSalesStageInfo* to obtain the key information stored on the blockchain, which contains the EA of enterprise and the Merkle Tree root stored by the enterprise. Finally, consumers check the Merkle Tree root for consistency and verify whether the enterprise that sells agricultural products to him is a legally registered enterprise by calling function *UserExists()* in the Management Contract.

### 4.4. The Design of Smart Contract 

A smart contract is a type of computer program that runs on the blockchain and can be executed automatically when certain conditions are satisfied. The agricultural traceability proposed in this paper is based on the Ethernet platform, and we use the Solidity language to write smart contracts. Through smart contracts, the system records the traceability information of agricultural products, tracks the status of agricultural products, and manages the agricultural supply chain enterprises. Table 4 presents the main functions of the smart contracts in the proposed system.

Algorithm 1 elaborates on the process of enterprise user registration. This function can only be called by the government regulator, and when the enterprises in the agricultural supply chain submit the required qualification information, the government regulator can complete the enterprise registration through this function after review. This function requires two parameters: one is the type of registered enterprise (production enterprise, processing enterprise, logistics enterprise, or sales enterprise), and the other is the registered enterprise’s Ethereum Address.
**Algorithm 1 Enterprise Register****Input:** EnterpiseType, EnterpriseEA**Output:** An event declaring the enterprise has been registered**Data:** EnterpiseType is the type of agricultural supply chain enterprise EnterpriseEA is the Ethereum Address of the enterprise to be registered1.**if** *FunctionCaller is not Government Regulator***then**2.  Display an error notification “Only regulator can operate!”3.**end**4.**else**5.  **if** *EnterpriseType is.ProductionEnterprise* **then**6.    producers[addr] = true7.  **else if** *EnterpriseType is ProcessingEnterprise* **then**8.    processors[addr] = true9.  **else if** *EnterpriseType is LogisticEnterprise*
**then**10.    logistics[addr] = true11.  **else if** *EnterpriseType is SalesEnterprise* **then**12.    seller[addr] = true13.  **end**14.**end**

Algorithm 2 elaborates on the process of banning or suspending an enterprise account. If an enterprise gives incorrect traceability information to consumers or has quality concerns with its products, the government regulator can call this function to block the enterprise’s account. This function requires two parameters: one is the enterprise’s Ethereum address, and the other is the type of enterprise.
**Algorithm 2 Ban Enterprise****Input:** EnterpiseType, EnterpriseEA**Output:** An event declaring the user has been banned**Data:** EnterpiseType is the type of agricultural supply chain enterprise EnterpriseEA is the Ethereum Address of the enterprise to be banned1.**if** *FunctionCaller is not Government Regulator***then**2.  Display an error notification “Only regulator can operate!”3.**end**4.**else**5.  **if** *EnterpriseType is.ProductionEnterprise* **then**6.    producers[addr] = false7.  **else if** *EnterpriseType is ProcessingEnterprise* **then**8.    processors[addr] = false9.  **else if** *EnterpriseType is LogisticEnterprise*
**then**10.    logistics[addr] = false11.  **else if** *EnterpriseType is SalesEnterprise* **then**12.    seller[addr] = false13.  **end**14.**end**

Algorithm 3 elaborates on the process of uploading traceability information by the agricultural supply chain enterprise. The input parameters of this function are the ID of the product, the constructed Merkle Tree root, and the type of enterprise. The function needs to meet the following two conditions to execute successfully: 

Firstly, the enterprise that calls the function is the same as the enterprise corresponding to this ID agricultural product. For example, the EA of the processing enterprise recorded in the blockchain for this ID agricultural product is *addr*. Then, only the processing enterprise with EA *addr* can call this function. Secondly, the supply chain link corresponding to the enterprise matches the current state of the agricultural products. For example, if the status of the agricultural product is *ProductionStage*, then only the traceability information of the production link can be uploaded at this time.
**Algorithm 3 Upload Traceability Information****Input**: EnterpiseType, ID, Merkle tree root**Output**: An event declaring the traceability information has been uploaded.**Data**: EnterpiseType is the type of agricultural supply chain enterprise ID is the ID of the agricultural product Merkle tree root is the root node of the Merkle tree constructed by traceability information1.**if** *FunctionCaller is not Products[ID].enterpriseAddr***then**2.  Display an error notification “You do not have permission for this product!”3.**else if** *EnterpiseType don’t match Products[ID].States***then**4.  Display an error notification “Status match error!”5.**else if** *EnterpriseType is ProductionEnterprise* **then**6.  Products[ID]. ProductionHash = Merkle tree root7.**else if** *EnterpriseType is ProcessingEnterprise* **then**8.  Products[ID]. ProcessingHash = Merkle tree root9.**else if** *EnterpriseType is LogisticsEnterprise* **then**10.  Products[ID]. LogisticsHash = Merkle tree root11.**else if***EnterpriseType is SalesEnterprise* **then**12.  Products[ID]. SalesHash = Merkle tree root13.**end**

## 5. Implementation and Evaluation

In this section, we discuss the implementation details and provide the cost, performance, and security analysis of the proposed system.

### 5.1. Implementation Details

In this subsection, we use the Remix IDE in-browser development and testing environment to test and validate key functions of the smart contracts. The Remix IDE, which is an open source web and desktop application, is used to compile and test the smart contracts within the private Ethereum blockchain [48]. Remix IDE produces logs for each transaction, which offer details about the transaction output, triggered events, and gas cost. Additionally, Remix IDE can perform syntax checking, runtime error messages, as well as customizable error messages by the developer, which helps the developer to debug the code to fix errors. 

To evaluate the functionality of our smart contracts, we deploy Management Contract, Production Contract, Processing Contract, Logistics Contract, and Sales Contract. Table 5 shows the Ethereum addresses of some stakeholders in the smart contracts. We further present the transactions and logs of the main smart contract’s functions below. 

**Figure A1:**
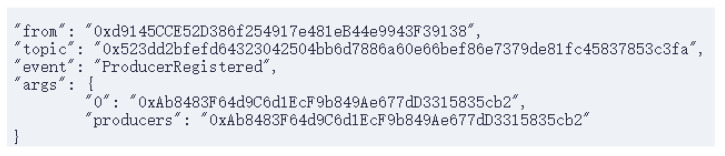
Successful execution of UserRegister Function.

**Figure A2:**
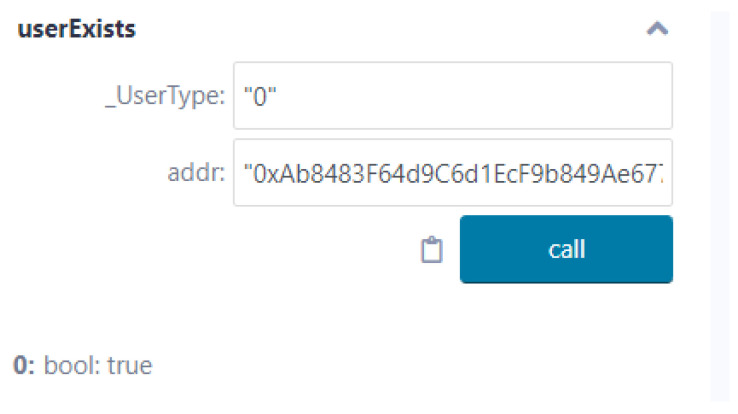
Successful execution of UserExists Function.

**Figure A3:**
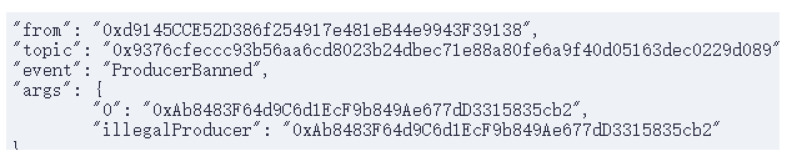
Successful execution of UserForbidden Function.

The *UserRegister* function is the most important key function in the Management Contract. In this function, it was tested whether only government regulators can register agricultural supply chain enterprises. The successful execution and its corresponding logs and events are displayed in Figure A1. The *UserExists* function in the Management Contract tested whether the address of the enterprise is a registered user in the traceability system. We enter the Ethereum address of the registered production enterprise and the results of the execution is are displayed in in Figure A2. *UserForbidden* function was tested government regulator block the enterprise’s account if the enterprise provides false traceability information or produce substandard agricultural products. The successful execution and its corresponding logs and events are displayed in Figure A3. 

**Figure A4:**
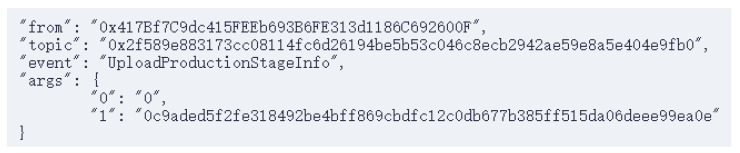
Successful execution of UploadProductionStageInfo Function.

**Figure A5:**
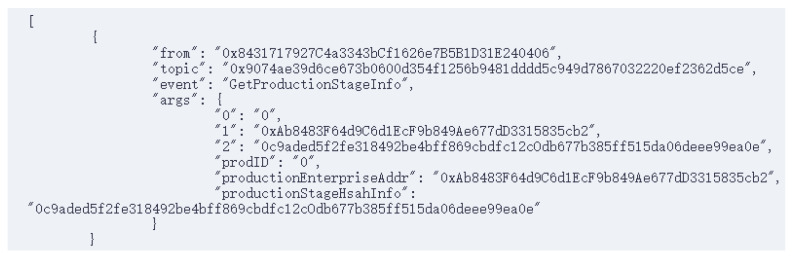
Successful execution of GetProductionStageInfo Function.

The *UploadProductionStageInfo* function was tested. The production enterprise stores the Merkle Tree root at the production link. The successful execution and its corresponding logs and events are displayed in Figure A4. There are two parameters in event “*UploadProduction*”: the first parameter is the ID of the agricultural product, and the second parameter is the Merkle Tree root of the traceability information at production link. *GetProductionStageInfo* was tested that a consumer enters the ID of an agricultural product to obtain the Ethereum address of the production enterprise and the Merkle Tree root constructed from the traceability information at the production link. The successful execution and its corresponding logs and events are displayed in Figure A5.

### 5.2. Cost Analysis

The user who calls functions in Ethereum smart contracts needs to pay a transaction fee measured in units of gas. “Gas” refers to the cost necessary to perform a transaction on the Ethereum blockchain [49]. Ethereum uses the mechanism of gas to control the number of resources that a transaction can use since it will be processed on thousands of computers around the world. The cost of calling a function is determined by the function, and the gas price set by the caller [50]. The amount of gas spent by each function depends on the complexity of the function itself, such as the number of function parameters, the execution steps of the function, etc. The price of gas is determined by miners depending on supply and the demand for the network’s computational power [51]. Each user can set the price of gas when calling a function, and miners will package and publish orders in accordance with the price of gas [52]. In other words, the higher the fee paid, the faster the corresponding transaction will be confirmed. 

Since the gas price is not a fixed value, the Ethereum Gas Station [53] provides information on the current prices of gas and live statics on how quickly transactions will be processed based on the gas price. According to the Ethereum Gas Station, the gas prices assumed on 15 March 2022 were 16, 19, and 25 Gwei, which respectively represented the gas prices for slow transactions (about 5 min), average transactions (about 5 min), and fast transactions (about 2 min). We use the conversion rate of ethers to USD of 256 in this analysis. Table 6 presents the gas cost of different function calls and their corresponding costs in US dollars (USD). The cost of any function does not exceed USD 0.287 for a slow transaction, USD 0.341 for an average transaction, and USD 0.448 for a fast transaction.

### 5.3. Performance Analysis

In the proposed traceability system, we adopt a data privacy protection solution by leveraging the hash function and the Merkle Tree. In this subsection, we test and analyze the performance of this data privacy protection solution. The Experimental environment is shown in Table 7.

In our solution, we first need to compute a hash of each piece of data in the traceability information. Table 8 shows the time for hashing data of different sizes using the SHA256 hash function. We can see that even for a data size of 1,000,000 KB (about 10 GB), it takes only about 2037 ms. Then, the computed hash value constitutes the leaf nodes of the Merkle Tree. To estimate the computational costs for constructing a Merkle Tree, we assume there are 10, 100, 1000, 10,000, and 100,000 pieces of data to be processed, respectively. Table 9 shows the time it takes to construct a Merkle Tree with a different number of leaf nodes. We can see from the table that even with up to 1,000,000 leaf nodes, it takes only about 931 s to construct a Merkle Tree. Therefore, our solution not only protects the enterprise’s private and sensitive data from being leaked but is also very efficient, which may be practical.

**Figure 9:**
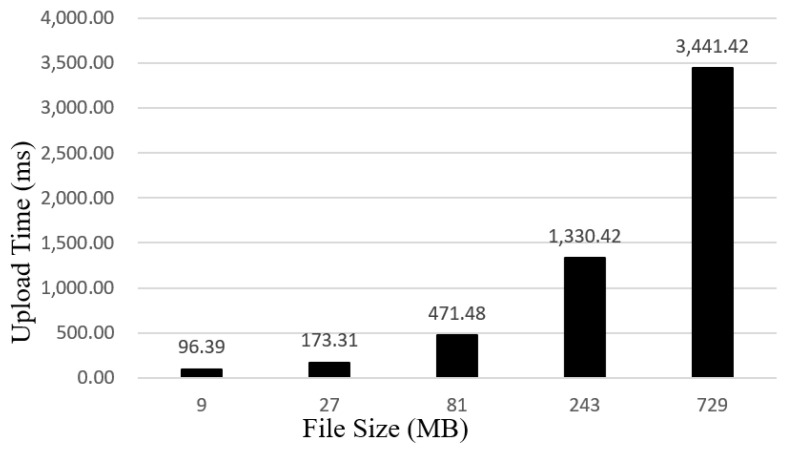
The relation between file size and upload time.

**Figure 10:**
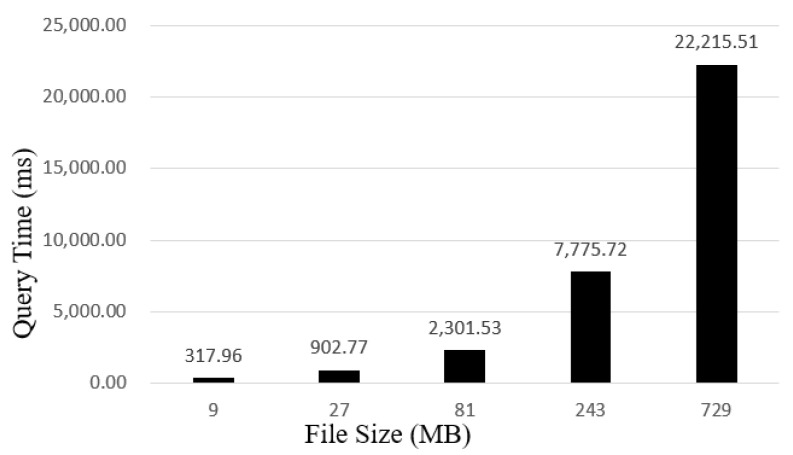
The relation between file size and query time.

The proposed traceability system adopts the dual storage model, and all attributes of traceability information of agricultural products at different links in the supply chain are separately stored off-chain in IPFS. As can be seen from Figure 9 and Figure 10, we tested the time consumed for uploading and downloading files of sizes 9, 27, 81, 243, and 729 MB, respectively. From the experimental results, it takes around 22 s to query a 243MB file and approximately 3.4 s to upload it. Therefore, enterprises and consumers can use this system to effectively record and query the detailed data of agricultural products in production, processing, logistics, and sales links. 

### 5.4. Security Analysis

In this subsection, we briefly discuss the security analysis of the proposed agricultural traceability system.

First, the main goal of the proposed system is to keep track of all information and transactions that occur within the agricultural product supply chain, ensuring agricultural product traceability. This goal is ensured in the proposed system because all traceability information and transaction records of agricultural products are stored in the immutable blockchain ledger. Second, the proposed system adopts the dual storage model of “Blockchain+IPFS”, in which large-size information is stored off-chain and small-size key information is stored on-chain. Because both the Ethereum Blockchain and IPFS are decentralized platforms that do not require a central server, even if the system receives malicious attacks such as a Denial of Service (DoS) attack, all functions are still available.

Third, the proposed system uses the modifier features of the Ethereum smart contract to qualify the execution conditions of each function. Therefore, all function callers can be traced, and they are accountable for their actions. Finally, it is critical to protect the traceability information against forgeries in the agricultural product supply chain. In our system, only enterprises authorized by government regulators are granted access to critical functions. Additionally, the system uses smart contracts to ensure that the traceability information of each agricultural product can only be uploaded by the designated enterprise and cannot be modified once uploaded.

## 6. Discussion

### 6.1. System Comparison

We compare the proposed system to the traditional agricultural product traceability system and related work in this subsection. The comparison between this system and the traditional agricultural traceability system is shown in Table 10, and the detailed analysis is provided as follows.

First, traditional agricultural product traceability systems are centralized in management and are typically managed by enterprises themselves, which means that issues such as single-point system failure, data tampering, and system maintenance difficulties are easily encountered [6]. In contrast, our proposed system is decentralized in nature, with all blockchain nodes worldwide maintaining the data in a collaborative manner. Second, traditional agricultural product systems store traceability information in their respective local databases, which is prone to data loss and the formation of “information islands.” [7]. This paper proposes a dual storage model in which the complete traceability information is stored off-chain in the distributed database IPFS, while the key information is stored on-chain in the blockchain, ensuring that the data are not easily lost and remain authentic. Third, due to the centralized management of traditional agricultural product traceability systems, driven by interests, enterprises may have the problem of unauthorized change of agricultural product traceability information, which can easily lead to information falsification. At the same time, the centralized database is easily attacked by the network. In this paper, we propose a decentralized agricultural traceability system using blockchain technology, where any data stored in the blockchain cannot be tampered with. Therefore, the reliability of traceability in this system can be guaranteed. Finally, it is more tedious and difficult to audit the traditional agricultural product traceability system in the event of agricultural product safety accidents. Blockchain is a decentralized distributed ledger where all users’ operations and transactions will be recorded and cannot be tampered with, and no user can deny the operation initiated by it once the transaction is completed. So, the proposed system can easily track and audit the behavior of everyone who takes part.

The proposed agricultural product traceability in this paper is compared with other related works. Table 11 gives the results of this comparison. The detailed analysis is as follows: First, the proposed system achieves traceability with trusted information in the entire agricultural product supply chain, which enables consumers to locate the source and verify the product’s quality. However, some references cannot realize this function [42]. Second, we select Ethereum platform to build the blockchain environment, and use the Solidity language to write the smart contract. Ethereum has a rich ecosystem of applications, which can help the agricultural product traceability system achieve greater value. It is noticed that other blockchain platforms, such as Hyperledger, can both realize the demand for agricultural product traceability [44]. Third, storing large amounts of data on-chain can be rather costly. The proposed system adopts a dual storage model that off-chain stores large-size data on IPFS, which can alleviate the data explosion issue of blockchain. However, some references store all data in the blockchain, which may increase the load pressure of the blockchain and influence the efficiency of the system [41,42,46,47]. Fourth, the traceability information contains enterprise privacy data that only regulators or relevant enterprises can view. The proposed system provides a data privacy protection solution by leveraging some cryptographic primitives and the Merkle Tree and can avoid the disclosure of enterprises’ sensitive data, which is essential in practical applications. However, some related works may lack privacy protection for traceability information [41,42,43,44,45,46]. Finally, we implement and test the proposed agricultural product traceability system to demonstrate the feasibility of our system. Additionally, we conducted a specific performance evaluation of the proposed system. We then discussed the overhead analysis of each action and transaction.

### 6.2. Limitations and Future Research Directions

The proposed agricultural product traceability system is implemented on the Ethereum Mainnet. Any form of transaction in Ethereum Mainnet needs to pay a gas fee. If the price of Ethereum becomes very high, then the cost of the traceability system will rise significantly, which is the main limitation of our current research. In our future work, we will conduct further research and plan to build an efficient and low-cost permissioned blockchain using an improved PoA (Proof-of-authority) consensus algorithm to solve the cost problem of the traceability system. Additionally, the proposed traceability system is less functional, and other technologies, such as RFID and artificial intelligence, could be combined in the future to make it more comprehensive and efficient.

## 7. Conclusions

In recent years, agricultural product safety accidents have raised public concern, jeopardizing people’s dietary safety and health. In order to keep track of specific information through the entire supply chain, including the production, logistics, processing, and sales processes, as well as to quickly find and prevent agricultural product safety problems, it is important to build a trusted traceability system. Traditional centralized traceability systems exist with the issues of insecure data storage, low traceability reliability, and single-point attack vulnerability. Blockchain technology has the characteristics of being data tamper-proof, distributed, decentralized, and traceable, which makes it a promising technology for agricultural product traceability.

Therefore, we proposed an agricultural product traceability system based on the Ethereum Blockchain. In this paper, we focused on the storage capacity and scalability issues and privacy leakage, which are the main challenges the agricultural product traceability system faces. We designed a dual storage model that stores small-size key information on-chain in the blockchain and stores big-size traceability information off-chain in the InterPlanetary File System to alleviate the blockchain’s storage pressure and enable efficient information queries. Furthermore, we present a data privacy protection solution to avoid the leakage of sensitive enterprise data in the traceability information. We implemented and tested the proposed system and conducted detailed cost, performance, and security analysis. The results prove the feasibility of the proposed system. In addition, we compared the proposed system with prior literature. Our study can provide a meaningful reference for individual countries and institutions. In the future, we will optimize the consensus algorithm to improve the system throughput and improve the system efficiency.

## Figures and Tables

**Table 2:** On-chain storage format.

Key	Value
ID	The unique identification of agricultural product
ProductionAddr	The Production Enterprise Ethereum Address
ProductionHash	The Merkle Tree root at production link
ProcessingAddr	The Processing Enterprise Ethereum Address
ProcessingHash	The Merkle Tree root at processing link
LogisticsAddr	The Logistics Enterprise Ethereum Address
LogisticsHash	The Merkle Tree root at logistics link
SalesAddr	The Sales Enterprise Ethereum Address
SalesHash	The Merkle Tree root at sales link
ProductionState	The current state of agricultural product

**Table 3:** Off-chain storage and results.

Content	Results
*data*_1_, *Hash*_2_, *Hash*_[3,4]_	ipfs_hash_1
*Hash*_1_, *data*_2_, *Hash*_[3,4]_	ipfs_hash_2
*Hash*_[1,2]_, *data*_3_, *Hash*_4_	ipfs_hash_3
*Hash*_[1,2]_, *Hash*_3_, *data*_4_	ipfs_hash_4

**Table 4:** The main functions of smart contracts.

Function Name	Function Description
UserRegister	Agricultural products supply chain enterprise registration
UserExists	Check whether the enterprise is registered
UserForbidden	Government regulator block the enterprise’s account
CreateNewProduction	Production enterprise create a new agricultural product structure.
UploadProductionStageInfo	Production enterprise store the Merkle Tree root at the production link
GetProductionStageInfo	Query the Merkle Tree root at the production link
ProductionToLogistic	Production enterprise hand over the products to logistics enterprise
UploadLogisticsStageInfo	Logistics enterprise store the Merkle Tree root at the logistics link
GetLogisticsStageInfo	Query the Merkle Tree root at the logistics link
LogisticToProcess	Logistics enterprise hand over the products to processing enterprise
LogisticToSales	Logistics enterprise hand over the products to sales enterprise
UploadProcessStageInfo	Processing enterprise store the Merkle Tree root at the process link
GetProcessStageInfo	Query the Merkle Tree root at the process link
ProcessToLogistic	Processing enterprise hand over the products to logistics enterprise
UploadSalesStageInfo	Sales enterprise store the Merkle Tree root at the process link
GetSaleStageInfo	Query the Merkle Tree root at the process link
SaleToConsumer	Sales enterprise hand over the products to consumer

**Table 5:** The Ethereum address of each stakeholder.

Stakeholder	Ethereum Address
Government Regulator	0x5B38Da6a701c568545dCfcB03FcB875f56beddC4
Production Enterprise	0xAb8483F64d9C6d1EcF9b849Ae677dD3315835cb2
Processing Enterprise	0x4B20993Bc481177ec7E8f571ceCaE8A9e22C02db
Logistic Enterprise	0x78731D3Ca6b7E34aC0F824c42a7cC18A495cabaB
Sales Enterprise	0x617F2E2fD72FD9D5503197092aC168c91465E7f2
Consumer	0x17F6AD8Ef982297579C203069C1DbfFE4348c372

**Table 6:** Gas cost of Ethereum functions in USD.

Function Name	Gas Cost	Slow Execution	Avg. Execution	Fast Execution
UserRegister	55,089	0.226	0.269	0.354
UserExists	24,864	0.102	0.121	0.160
UserForbidden	26,220	0.108	0.128	0.168
CreateNewProduction	69,844	0.287	0.341	0.448
UploadProductionStageInfo	50,463	0.207	0.246	0.324
GetProductionStageInfo	25,027	0.103	0.122	0.161
ProductionToLogistic	50,010	0.205	0.244	0.321
UploadLogisticsStageInfo	50,233	0.206	0.245	0.322
GetLogisticsStageInfo	24,274	0.100	0.118	0.156
LogisticToProcess	48,746	0.200	0.238	0.313
LogisticToSales	50,175	0.206	0.245	0.322
UploadProcessStageInfo	50,143	0.203	0.238	0.309
GetProcessStageInfo	25,427	0.104	0.124	0.163
ProcessToLogistic	50,412	0.207	0.246	0.324
UploadSalesStageInfo	50,658	0.208	0.247	0.325
GetSaleStageInfo	24,765	0.102	0.121	0.159
SaleToConsumer	50,376	0.207	0.246	0.323

**Table 7:** Experiment Environment.

Type	Description
CPU	AMD Ryzen 7 4800H
GPU	RTX 2060
RAM	16 GB
SSD	512 GB
Operating System	Windows 10

**Table 8:** Hash Performance.

Date Size (kb)	Time (ms)
1	13
10	14
100	17
1000	25
10,000	57
100,000	282
1,000,000	2037

**Table 9:** The Performance of Constructing Merkle Tree.

Number of Leaf Nodes	Time (ms)
10	99
100	193
1000	1045
10,000	8963
100,000	931,263

**Table 10:** Comparison with Traditional Agricultural Traceability System.

Features	Traditional System	Our System
System Management	Centralization	Decentralization
Data Storage	Local Database	Blockchian + IPFS
Reliability of Traceability Results	Low	High
Auditability	Low	High

**Table 11:** Comparison of the proposed system to related works.

Research	Traceability	Blockchain Platform	Off-Chain Storage	Privacy Protection	Implementation	Performance Evaluation
[41]	✓	/	🗴	🗴	🗴	🗴
[42]	🗴	/	🗴	🗴	🗴	🗴
[43]	✓	Ethereum	✓	🗴	✓	✓
[44]	✓	Sawtooth	✓	🗴	✓	🗴
[45]	✓	Ethereum	✓	🗴	✓	🗴
[46]	✓	/	🗴	🗴	✓	✓
[47]	✓	/	🗴	✓	✓	✓
This paper	✓	Ethereum	✓	✓	✓	✓

## Data Availability

Not applicable.
